# Endocytosis and Sphingolipid Scavenging in *Leishmania mexicana* Amastigotes

**DOI:** 10.1155/2012/691363

**Published:** 2011-09-21

**Authors:** Hayder Z. Ali, Clare R. Harding, Paul W. Denny

**Affiliations:** ^1^Biophysical Sciences Institute, Department of Chemistry and School of Biological and Biomedical Sciences, Durham University, Durham DH1 3LE, UK; ^2^School of Medicine and Health, Durham University, Queen's Campus, Stockton-on-Tees TS17 6BH, UK

## Abstract

*Leishmania* species are the causative agents of the leishmaniases, a spectrum of neglected tropical diseases. Amastigote stage parasites exist within macrophages and scavenge host factors for survival, for example, *Leishmania* species utilise host sphingolipid for synthesis of complex sphingolipid. In this study *L. mexicana* endocytosis was shown to be significantly upregulated in amastigotes, indicating that sphingolipid scavenging may be enhanced. However, inhibition of host sphingolipid biosynthesis had no significant effect on amastigote proliferation within a macrophage cell line. In addition, infection itself did not directly influence host biosynthesis. Notably, in contrast to *L. major*, *L. mexicana* amastigotes are indicated to possess a complete biosynthetic pathway suggesting that scavenged sphingolipids may be nonessential for proliferation. This suggested that Old and New World species differ in their interactions with the macrophage host. This will need to be considered when targeting the *Leishmania* sphingolipid biosynthetic pathway with novel therapeutics.

## 1. Introduction

*Leishmania* species are insect vector-borne kinetoplastid protozoan pathogens causing a wide spectrum of neglected tropical diseases (the leishmaniases—cutaneous, mucocutaneous, and visceral) in humans across the globe. Moreover, the spread and severity of disease are exacerbated by its status as an important coinfection in AIDS patients [1]. *Leishmania *species exhibit a digenetic lifecycle: (a) existing as flagellated promastigotes within the sand fly vector; (b) following an insect bite and uptake by professional phagocytic cells, especially macrophages, differentiating into the aflagellate amastigote form. Within the host macrophage the parasite proliferates within acidic, fusogenic, endosome-derived phagolysosomes [2]. This compartment intersects with the phagosomal and autophagosomal pathways meaning that the parasites have access to the rich mix of nutrients resulting from degradation of phagocytosed macromolecules within the lytic environment of the phagolysosome. However, residency within such a compartment could also lead to contact with components of the host major histocompatibility complex (MHC), subsequent macrophage activation, and parasite killing. To overcome this certain species of *Leishmania *sequester and degrade MHC molecules [3, 4]; presumably this occurs via endocytosis although the mechanism has not been characterised. Endocytosis is also the process by which nutrients can be taken into the parasite, for example, *L. amazonensis* amastigotes were reported to endocytose transferrin from the phagolysosome [5]. However other species, including *L. mexicana, *do not show this behaviour [6]. Low-density lipoprotein [7] and haemoglobin [8] have also been shown to be endocytosed by *Leishmania *promastigote forms. 

Sphingolipids are amphipathic lipids consisting of a sphingosine backbone with a long-chain fatty acid and a polar alcohol as attachments. Ceramide is an unmodified sphingolipid that functions as a secondary messenger in ubiquitous, eukaryotic signalling mechanisms. Modified (complex) sphingolipids are major components of the outer leaflet of eukaryotic plasma membranes believed to be involved, with sterols, in the formation of microdomains or lipid rafts. Rafts are proposed to function in a diverse array of processes from the polarised trafficking of lipid-modified proteins, to the assembly of signal transduction complexes [9]. The first, rate-limiting, enzyme in sphingolipid biosynthesis is serine palmitoyltransferase (SPT). SPT catalyses the condensation of L-serine and palmitoyl CoA to form 3-ketodihydrosphinganine. Subsequently, *N*-acetylation of sphingoid base in the endoplasmic reticulum (ER) leads to the formation of ceramide, which is converted to complex sphingolipids (e.g., sphingomyelin SM or inositol phosphorylceramide—IPC) via sphingolipid synthases. The animal sphingolipid synthase, SM synthase (SMS), transfers phosphorylcholine from phosphatidylcholine (PC) to ceramide to give SM; in contrast yeast, plants, and the kinetoplastid protozoan parasite *Leishmania* utilise IPC synthase to catalyse the formation of IPC via the transfer of phosphorylinositol from phosphatidylinositol (PI) to (phyto)ceramide [10]. This non-mammalian enzyme has long been established as a drug target for antifungals [11], and more recently, the sphingolipid synthase from *Trypanosoma brucei* (a kinetoplastid relative of *Leishmania*) has been demonstrated to be essential [12, 13].

Intriguingly, biosynthesis of sphingoid base and ceramide, precursors of complex sphingolipids, is absent and nonessential for the proliferation of* L. major *amastigotes within phagolysosomes in a model animal system [14, 15]. However, intramacrophage amastigotes synthesize the complex sphingolipid IPC *de novo* utilising precursors from alternative, host sources and maintain this lipid species at equivalent cellular levels to promastigotes [15]. It has been shown that *L. donovani* stimulate host macrophages to upregulate the production of ceramide, a substrate for IPC synthase [16] and a downstream product of SPT. Furthermore, recent work has identified the *L. major *SMase (*Lmj*ISCL) and shown it to be essential for pathogenicity in an animal model. This indicated that the generation of ceramide from host SM, via *Lmj*ISCL, is key to proliferation within the phagolysosome [17, 18]. Taken together, these studies indicated that *L. major *(and perhaps *L. donovani, *another Old World species) scavenges host sphingolipids to generate ceramide for amastigote proliferation, pathogenicity, and IPC biosynthesis.

Like amastigote *Leishmania, *the bacterial pathogen *Coxiella burnetti* (the aetiologic agent of Q fever) replicates within an acidified endocytic compartment and scavenges the sphingolipid SM from the plasma membrane via endocytosis [19]. In this work we sort to establish whether *Leishmania *employs a similar strategy to acquire and utilise essential host sphingolipids.

## 2. Materials and Methods

### 2.1. Cell Culture

*Leishmania mexicana *(MNYC/BZ/62/M379) promastigotes were maintained at 26°C in Schneider's media (Sigma Aldrich) pH7, supplemented with 15% foetal bovine sera (FBS, Biosera Ltd). Promastigotes were differentiated to amastigote forms in Schneider's media with 20% FBS at pH 5.5 and 32°C according to the published protocol [20]. The continuous murine macrophage cell line RAW264.7 was maintained in DMEM (Gibco-BRL) with 10% FBS, at 37°C and 5% CO_2_. The cytoxicity of myriocin (Sigma Aldrich) was established using the AlamarBlue (Invitrogen) assay according to manufacturer's protocol and as previously [21, 22]. The efficacy of myriocin was confirmed using a yeast diffusion assay [23].

### 2.2. Metabolic Labelling

*L. mexicana* axenic promastigotes and amastigotes (10^7^ mL^−1^) were incubated in serum-free Schneider's media for 30 minutes before labeling in the same with 5 *μ*M of BSA conjugated BODIPY FL C_5_-ceramide (Invitrogen) at 26°C and 32°C, respectively, for 2 hours. Promastigote parasites were similarly labeled for 16 hours in DMEM (ICN) supplemented with 10% FCS (Gibco BRL) and 20 mCi mL^−1^ [myo-^3^H]-inositol (102 Ci mmol^−1^ Amersham) [14]. For serine-containing lipid analysis axenic amastigotes (5 × 10^7^ mL^−1^) were incubated for 45 minutes in MEM Eagle (Sigma Aldrich) then labeled for 8 hours in the same medium containing 20 mCi mL^−1^ [^3^H]-L-serine (20 Ci mmol^−1^; ICN) [14]. Lipids were extracted and analysed as previously described [23].

### 2.3. Endocytosis Assay

*L. mexicana *cells were washed three times using warm serum-free media and counted using a Neubauer haemocytometer. 10^7^ cells were then incubated with 50 *μ*gmL^−1^ 3 k Texas Red Dextran (Invitrogen) in 500 *μ*L of serum-free media for 2 hours at 32°C. Controls were incubated on ice. Cells were subsequently washed 5 times with ice cold PBS and fixed with 3.7% formaldehyde. After washing, cells were resuspended in 500 *μ*L of PBS and the fluorescence quantified using a plate reader (FLx800TM, BioTek; 590/20 Ex 645/40 Em). Observation of cells by fluorescence microscopy indicated that the dextran had been taken up by the parasites.

### 2.4. Macrophage Infection

1 × 10^5^ RAW264.7 murine macrophages in DMEM were allowed to adhere to coverslips within each well of a 24-well tissue culture plate (Nunc) and then incubated for 24 hours in appropriate media (DMEM with 10% FBS or with 1% Nutridoma, Roche) with or without myriocin. *L. mexicana* amastigotes were then applied at a ratio of 10 : 1 in media and the infection allowed to proceed for 48 hours at 32°C and 5% CO_2_, with daily changes of media with myriocin where appropriate. 

### 2.5. Expression Analyses

Denatured parasite lysates were separated and immunoblotted as described [24] and the filters probed with mouse anti-*Lmj*LCB2 or rabbit anti-*Lmj*NMT polyclonal primary antibodies [14] at 1 : 1000, followed by horseradish peroxidase-conjugated secondary antibodies (Sigma-Aldrich). Complexes were detected using the ECL system (Amersham Pharmacia). For the mRNA analyses, total RNA from equivalent numbers of 48 hours infected, or noninfected, RAW264.7 cells were extracted using the RNeasy kit (Qiagen) according to the manufacturer's protocol. Following DNase treatment (RQ1, Promega) cDNA was synthesized using the ImProm-II Reverse Transcription System (Promega) according to manufacturer's protocol. Quantitative PCR was performed in a Rotor-Gene RG3000 (Corbett Research) using SYBR Green Jump-Start Taq Ready Mix (Sigma Aldrich) according to the manufacturer's instructions. The murine *MmLcb2* (encoding subunit 2 of SPT) was amplified using the primer pair-5′AGGTGGATATCATGGAGAGA 3′ and 5′GATCCAGTGTTCCTCGC 3′. The reference gene, *MmCasc3, *was amplified using primers as previously [25]. The qPCR was carried out in triplicate on 3 replicates with annealing temperature 52°C for *MmLcb2 *and 55°C for *MmCasc3*.

## 3. Results and Discussion

### 3.1. Endocytosis in Promastigote and Amastigote *L. mexicana*

Like *Leishmania *species, *Trypanosoma brucei *(which causes the neurological NTD African sleeping sickness) is a kinetoplastid parasite. However, whereas the pathogenic amastigote forms of *Leishmania* species shelter within macrophages, the mammalian form of *T. brucei* proliferates extracellularly within the bloodstream of the host. Here it must avoid the full force of the immune system, and the switching of the predominant surface molecule, Variable Surface Glycoprotein (VSG), is key to this. Endocytosis of VSG is an important part of this antigenic variation and immune evasion. Notably, the rate of endocytosis in bloodstream form (BSF) *T. brucei* is 10 times higher than in the insect stage procyclic form (PCF), and the entire VSG coat is replaced every 7 minutes [26]. This allows the internalisation and degradation of bound antibodies and complement factors which helps protect the free-living parasite from the humoral immune response. This upregulation of endocytosis is facilitated by an increase in expression of factors involved in the uptake of VSG and its subsequent trafficking and processing [27, 28]. Before this study it remained unknown whether *Leishmania *species increased their rate of endocytosis on differentiation to the mammalian amastigote form. This may be predicted to facilitate the observed uptake of MHC and immune avoidance [6] or allow the acquisition of host sphingolipid for use in IPC synthesis [15, 18].

**Figure 1:**
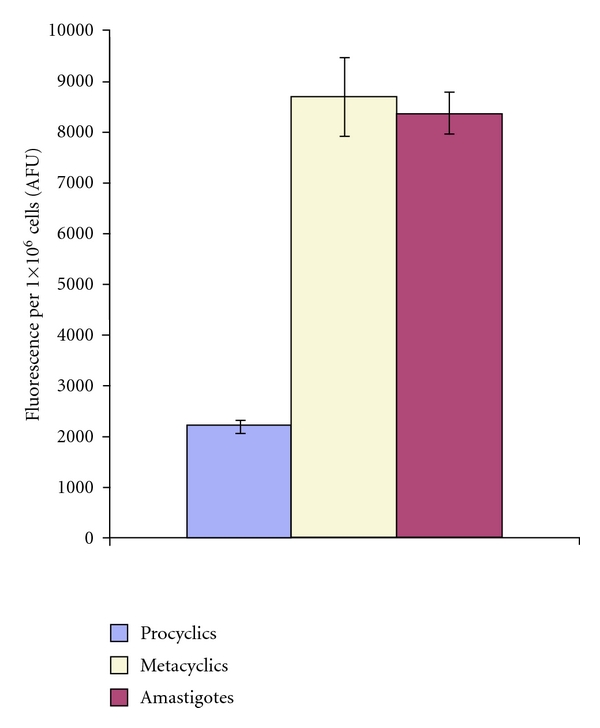
The endocytosis of Texan Red labelled dextran at 32°C measured as described and with the background (uptake in control parasites at 0°C) subtracted. Results from a representative experiment, in triplicate with standard deviation shown. AFU: arbitrary fluorescence units.

To address this the New World species *L. mexicana* was employed. Unlike *L. major, *this species can be grown in both promastigote and amastigote stages in axenic cell culture [20] allowing direct comparative analyses to be undertaken [29]. To quantify endocytosis fluorescently labelled dextran was utilised. Previously, dextran has been demonstrated to be taken up, via the endocytic pathway, by *L. donovani* promastigotes [30]. Here, axenic cell equivalents of promastigotes (both noninfective, proliferating procyclics, and infective, nonproliferating metacyclics) and amastigotes were labelled under identical conditions (32°C in serum-free media). Dextran is measurably taken up by procyclic promastigotes; however the quantity endocytosed by amastigotes is more than 4 times greater (Figure 1). Interestingly the nondiving metacyclic forms demonstrate a similar up-regulation of this activity.

These data indicate that endocytosis is up regulated in amastigote forms, and this could be speculated to facilitate efficient immune evasion or substrate scavenging as discussed. The observation of similar levels of endocytic activity in metacyclic promastigotes was surprising given their apparent quiescence [20]. However, one may speculate that this indicated that the machinery for this process is acquired at this stage ready for the establishment of amastigote infection in a mammalian host.

### 3.2. The Role of Host Sphingolipid Biosynthesis in *L. mexicana* Invasion and Proliferation

**Figure 2:**
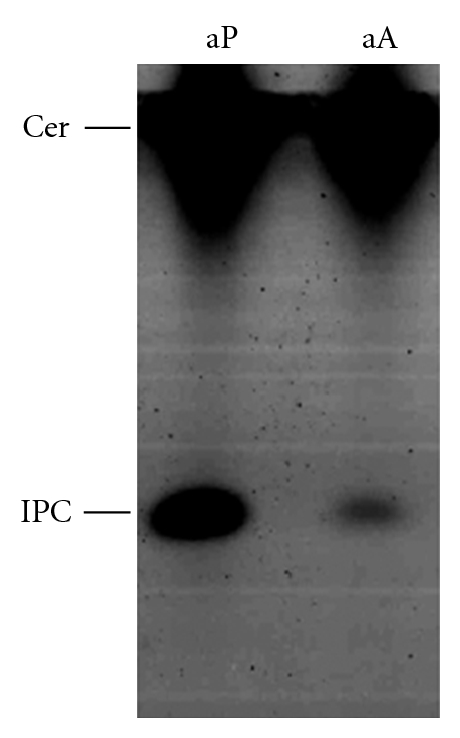
HPTLC analysis of axenic procyclic promastigotes (aP) and amastigotes (aA) metabolically labelled with BODIPY FL C_5_-ceramide. IPC: inositol phosphorylceramide; Cer: ceramide.

To investigate IPC synthase activity in *L. mexicana*, axenic promastigotes and amastigotes were metabolically labeled with fluorescent BODIPY FL-ceramide (Figure 2). Both cell types produced a labelled product that comigrated with IPC, although the level in amastigotes was relatively low reflecting the slower growth of this form. These data indicated that *L. mexicana *has an active IPC synthase in both lifecycle stages, as does *L. major* [15].

As discussed above, host sphingolipid must be acquired as a source of ceramide substrate for the IPC synthase of amastigote *L. major *[15]. In support of this several studies have identified host cell glycosphingolipids in intramacrophage amastigotes of *Leishmania *species [31–33]. In addition, it has been reported that fumonisin B1 (an inhibitor of dihydroceramide synthase and ceramide synthesis) inhibits the replication of intramacrophage *L. donovani* [34]. Utilising myriocin (a potent, specific SPT inhibitor [35]) the role of host synthesized sphingolipids was investigated in *L. mexicana *infected RAW264.7 cells, a continuous murine macrophage cell line. The viability of axenic promastigotes and amastigotes (data not shown) was unaffected by the presence of 50 *μ*M myriocin as previously reported [36]. Similarly, the RAW264.7 host cells were viable at 50 *μ*M myriocin; levels above this lead to detachment of cells from the tissue culture well (data not shown).

**Figure 3:**
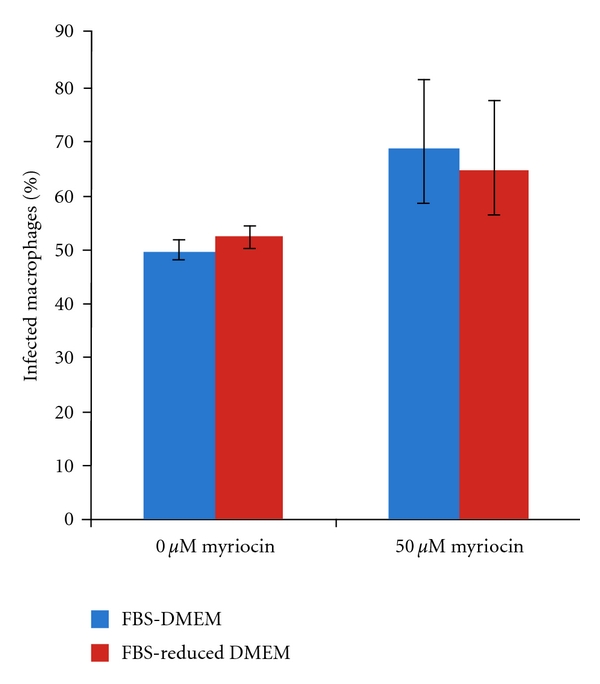
Invasion, % infected macrophages, determined 48 hours after infection in the presence or absence of the SPT inhibitor myriocin and with (FBS-DMEM) or without (FBS-reduced DMEM) exogenous serum in the media. Results of three independent experiments.

**Figure 4:**
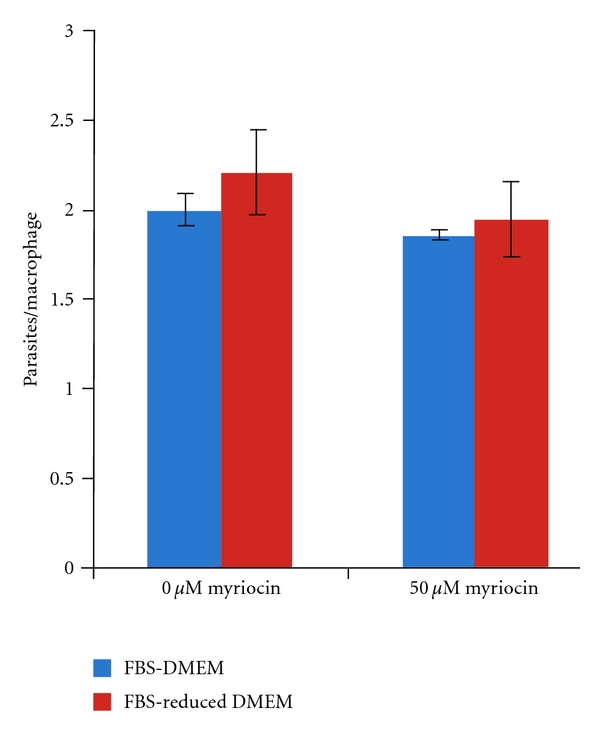
Proliferation, parasites per infected macrophage, determined 48 hours post infection in the presence or absence of the SPT inhibitor myriocin, and with (FBS-DMEM) or without (FBS-reduced DMEM) exogenous serum in the media. Results of three independent experiments.

In this study 50 *μ*M of myriocin was applied to RAW264.7 cells for 24 hours prior to challenge with *L. mexicana *axenic amastigotes at a ratio of 10 : 1. With daily application of the drug the infection was allowed to continue for 48 hours prior to fixation, staining, and counting as described in Section 2. This experiment was conducted either in the presence of full (10%) serum or in serum-reduced (1% Nutridoma) media. The latter conditions reduce the quantity of exogenous sphingolipid available to the RAW264.7 cells and the intracellular parasites. Under both sets of conditions the proportion of macrophages infected with amastigotes is unaffected by the presence of myriocin (Figure 3), as is the average number of parasites per host cell (Figure 4). These data indicate that host sphingolipid biosynthesis and exogenous sphingolipids are not central to the invasion and proliferation of *L. mexicana *in RAW264.7macrophages.

Similarly, myriocin has been reported not to inhibit *L. major *amastigote replication in cultured murine macrophages [15]. In addition, unlike in *L. donovani* [34], the specific ceramide synthase inhibitor fumonisin B1 had no demonstrable affect on the proliferation of intra-macrophage *L. major *amastigotes [15]. Together, these results indicated that host sphingolipid synthesis is not central to the proliferation of *L. major* or *L. mexicana *amastigotes within macrophages. However, it remains possible that free ceramide is generated by the host via complex sphingolipid catabolism—a process up regulated in *L. donovani* infected macrophages [16]. In this study the macrophages were preincubated for 24 hours with myriocin in the absence of serum in order to deplete the host sphingolipid available for parasite scavenging. However, inhibition of SPT for a length of time equivalent to the experimental conditions employed here only leads to partial (up to ~80%) depletion of complex sphingolipids in CHO cells [37]. This indicated that preformed complex sphingolipids are stably maintained in the cell membranes for some time after the inhibition of *de novo *synthesis. It is possible that such lipids could continue to be catabolised and scavenged by amastigote *L. mexicana.*

### 3.3. Host and Parasite Serine Palmitoyltransferase (SPT) Expression during Macrophage Infection

Notably host *de novo* sphingolipid synthesis is up regulated during *L. donovani* proliferation within macrophages [16], and it is possible that a similar effect during *L. mexicana* infection could mask the efficacy of the SPT inhibitor myriocin. To test the hypothesis that an increase in host sphingolipid biosynthesis, via the up regulation of biosynthetic enzymes, is elicited by the parasite, the levels of host *MmLcb2 *(encoding subunit 2 of SPT) transcript with and without *L. mexicana *infection were established using real-time qPCR. To do this normalisation to a reference was required; of nine commonly used reference genes in RAW264.7 cells only *Casc3* was previously found to be suitable and sufficient for this purpose on infection with *Mycobacterium avium *[25]. Normalised to *MmCasc3 *expression, *MmLcb2* mRNA levels were unchanged 48 hours after infection with *L. mexicana *(Table 1) under the conditions described. This indicated that host sphingolipid biosynthesis is not up regulated on infection.

**Figure 5:**
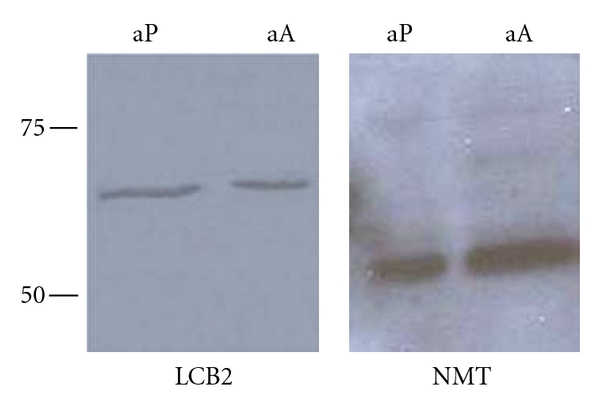
Lysates of 5 × 10^6^ axenic procyclic promastigotes (aP) and amastigotes (aA) probed with the cross reacting *Lmj*LCB2 and *Lmj*NMT antibodies in a Western blot. Molecular weight markers in kDa shown on left of image.

These results were perhaps surprising given the previously observed down-regulation of the *Lm*LCB2 protein in *L. major *amastigote forms [14, 38], coupled with the maintenance of IPC biosynthesis [15] and the requirement for catabolism of scavenged SM [17, 18]. In light of these data it was chosen to examine the expression profile of the *L. mexicana *SPT. Utilising the cross reactive anti-*Lmj*LCB2 antibody [14, 39] the expression of *Lmx*LCB2 was probed using Western blotting (Figure 5). The levels of the constitutively expressed *N*-myristoyltransferase (NMT) were used as a loading control [24].

**Figure 6:**
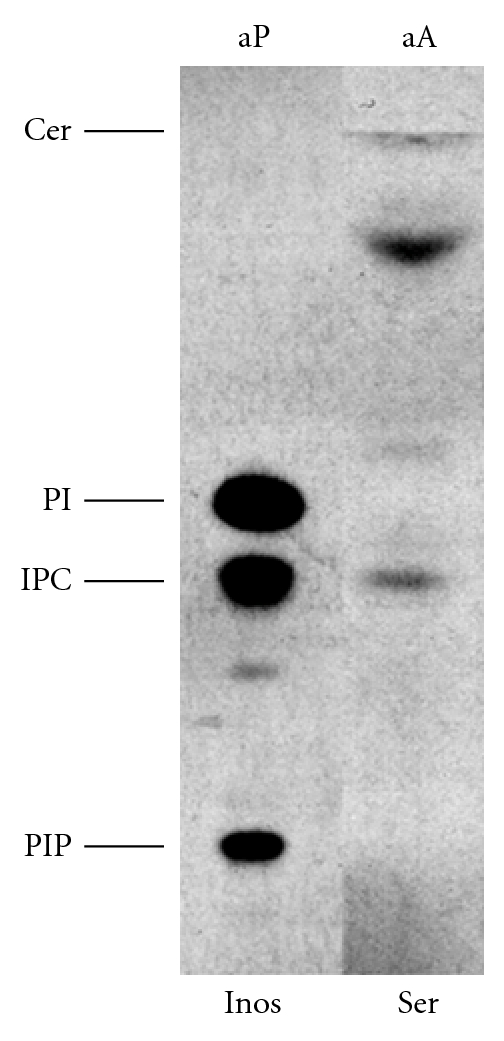
HPTLC analysis of axenic amastigotes (aA) metabolically labelled with tritiated serine (Ser; 1.25 × 10^7^ cell equivalent). Axenic promastigotes (aP) similarly labelled with tritiated inositol (Inos; 5 × 10^6^ cell equivalent) served as markers. Following fluorography the plate was exposed to film for 15 days. PIP: phosphatidylinositol phosphate; IPC: inositol phosphorylceramide; PI: phosphatidylinositol; Cer: ceramide, migrating at solvent front.

This indicated that *Lmx*LCB2, and so *Lmx*SPT, is constitutively expressed throughout the lifecycle of *L. mexicana.* These data have been recently confirmed by proteomic analyses of isolated, intra-macrophage *L. mexicana *amastigotes [40]. Furthermore, metabolic labelling of axenic amastigotes demonstrated the incorporation of tritiated serine into the primary complex sphingolipid, IPC (Figure 6).

Taken together, these data indicated that this New World species has a complete and active *de novo* sphingolipid biosynthetic pathway in the amastigote stage, and this contrasts with Old World *L. major * [14, 38]. In addition, the possibility that myriocin may inhibit *Lmx*SPT in intra-macrophage *L. mexicana *amastigotes needs to be considered in relation to the data shown in Figure 4. If this compound is able to access and inhibit the parasite enzyme in these assays, then the results obtained would imply that both host and parasite SPT activity are nonessential for *L. mexicana *proliferation, a similar scenario to that seen in *L. major * [15].

### 3.4. Summary

These data are the first to demonstrate that the endocytic rate of the pathogenic, amastigote stage of *Leishmania *species is raised when compared with insect stage procyclic promastigotes. This is reminiscent of the situation in *T. brucei*, where endocytosis is up regulated on differentiation to mammalian BSF parasites in order to facilitate immune evasion [36]. This dramatic adaptation is facilitated by an increase in expression of factors involved in the uptake of molecules and their subsequent trafficking and degradation [27, 28]. In *Leishmania* species proteolytic activity is highly up regulated during differentiation from promastigotes to amastigotes, and this coincides with the appearance of multivesicular lysosomes (megasomes) [41]. This correlates with the uptake of host MHC molecules [41] and, as shown here, an increase in endocytosis. However, unlike in *T. brucei,* none of the known molecular machinery of endocytosis so far studied in *Leishmania *species has demonstrated any increased expression on differentiation [24, 42]. However, a RAB-like GTPase in *L. major* has been shown to have increased expression at the level of mRNA in amastigote forms, although the role of this factor in the cell remains unknown [43].

Both promastigotes and amastigotes of *L. major* [15] and *L. mexicana *are able to synthesize the primary complex sphingolipid, IPC. Old World *L. major* is able to do this in the absence of *de novo *SPT activity, therefore is the observed increase in endocytic activity related to an ability to scavenge host sphingolipid for use as substrate [15]? The data presented here suggest that *L. mexicana* amastigotes can invade and proliferate normally when host sphingolipid biosynthesis is inhibited and/or when the exogenous source of lipid (serum) is removed. This suggested that the parasite is not wholly dependant on ongoing host sphingolipid synthesis, although residual host complex sphingolipid may remain and be sufficient for the pathogen. Analyses of host *MmLcb2 *and parasite *Lmx*LCB2 expression indicated that infection does not influence host sphingolipid biosynthesis and that promastigote and amastigotes stages of *L. mexicana* equivalently express SPT, the first enzyme in sphingolipid biosynthesis [40]. This is in contrast to *L. major *where *Lmj*LCB2 is down regulated and nonessential for pathogenesis and complex sphingolipid biosynthesis [14, 38]. This implies that there are profound differences between the Old and New World species in terms of the relationship between the *Leishmania *parasite and its mammalian host cell. Further knowledge of these differences will be required when considering the targeting of lipid biosynthesis for the development of novel therapies.

## Figures and Tables

**Table 1:** Expression of *MmLcb2* in infected and noninfected macrophages.

Serum	Normalised *MmLcb2 *infected: noninfected ± standard deviation
+	1.04 ± 0.12
−	0.94 ± 0.12
